# Alcohol Intoxication and Cognition: Implications on Mechanisms and Therapeutic Strategies

**DOI:** 10.3389/fnins.2020.00102

**Published:** 2020-02-12

**Authors:** Asha Jacob, Ping Wang

**Affiliations:** ^1^Center for Immunology and Inflammation, The Feinstein Institutes for Medical Research, Northwell Health, Manhasset, NY, United States; ^2^Department of Molecular Medicine, Donald and Barbara Zucker School of Medicine at Hofstra/Northwell, Hempstead, NY, United States; ^3^Department of Surgery, Donald and Barbara Zucker School of Medicine at Hofstra/Northwell, Hempstead, NY, United States

**Keywords:** binge alcohol drinking, cognitive impairment, positron emission tomography, object place memory task, animal model of binge alcohol

## Abstract

Binge alcohol drinking is highly prevalent in young adults and results in 30% deaths per year in young males. Binge alcohol drinking or acute alcohol intoxication is a risk factor for developing alcohol use disorder (AUD). Three FDA approved drugs are currently in use as therapy for AUD; however, all of them have contra-indications and limitations. Structural brain imaging studies in alcoholics have shown defects in the brain regions involved in memory, cognition and emotional processing. Positron emission tomography (PET) using radiotracers (e.g., ^18^FDG) and measuring brain glucose metabolism have demonstrated diagnostic and prognostic utility in evaluating patients with cognitive impairment. Using PET imaging, only a few exclusive human studies have addressed the relationship between alcohol intoxication and cognition. Those studies indicate that alcohol intoxication causes reduction in brain activity. Consistent with prior findings, a recent study by us showed that acute alcohol intoxication reduced brain activity in the cortical and subcortical regions including the temporal lobe consisting the hippocampus. Additionally, we have observed a strong correlation between reduction in metabolic activity and spatial cognition impairment in the hippocampus after binge alcohol exposure. We have also demonstrated the involvement of a stress response protein, cold inducible RNA binding protein (CIRP), as a potential mechanistic mediator in acute alcohol intoxication. In this review, we will first discuss in detail prior human PET imaging studies on alcohol intoxication as well as our recent study on acute alcohol intoxication, and review the existing literature on potential mechanisms of acute alcohol intoxication-induced cognitive impairment and therapeutic strategies to mitigate these impairments. Finally, we will highlight the importance of studying brain regions as part of a brain network in delineating the mechanism of acute alcohol intoxication-induced cognitive impairment to aid in the development of therapeutics for such indication.

## Introduction

Alcohol is the most widely used substance of abuse. While only 7% of the general population are heavy drinkers, close to half of the population consume alcohol on a regular basis. Excessive alcohol use is especially harmful to younger age group and it results in 30% deaths per year in males aged 15–29 years (Lopez-Caneda et al., 2018). Binge alcohol drinking is a popular mode of alcohol intake in young adults. The National Institute of Alcoholism and Alcohol Abuse (NIAAA) defines binge alcohol drinking as a pattern of consumption where the person’s blood alcohol concentration (BAC) reaches to 0.08% or above. This level of BAC is typically achieved when men consume 5 drinks or women consume 4 drinks within 2 h. Binge drinking negatively affects nearly all organ systems, especially the brain regions responsible for memory, coordination and emotional processing (Jacobus and Tapert, 2013). Prior structural magnetic resonance imaging (MRI) studies indicate that white matter development defect as well as systemically thinner and lower volumes in prefrontal cortex and cerebellar regions are observed among adolescent binge drinkers (Pfefferbaum et al., 2016). Subcortical regions including the hippocampus, diencephalon, cerebellum and brain stem also exhibit decreased volume among binge drinkers (Medina et al., 2007; Lisdahl et al., 2013; Squeglia et al., 2014). In alcoholics, studies in neuroimaging, neurophysiology, neuropathology and neuropsychology show that the frontal lobes, limbic system and cerebellum are particularly prone to damage and dysfunction (Oscar-Berman and Marinkovic, 2007). Functional imaging studies indicate that the anterior cingulate cortex, lateral prefrontal cortex and parietal brain regions, which are each implicated in cognitive control, are affected by acute alcohol intoxication (Soderlund et al., 2007; Anderson et al., 2011). Positron emission tomography (PET) which utilizes radiotracers (e.g., ^18^FDG, ^11^C-glucose) to measure brain glucose metabolism as a surrogate for brain activity, has shown that in alcoholics and in non-alcoholic human subjects neuronal activity was reduced after acute alcohol administration (Wik et al., 1988; Volkow et al., 1990, 2006; Bjork and Gilman, 2014). PET imaging of the brain with ^18^FDG has demonstrated diagnostic and prognostic utility in evaluating patients with cognitive impairment (Silverman et al., 2008). In alcoholics, the severity of the clinical neurological impairment significantly correlated with hypometabolism in distinct brain regions (Gilman et al., 1990). In this review, we will first describe the studies in detail on the correlation of PET imaging and cognitive assessment after alcohol intoxication. Next, we will review the existing body of literature on the potential mechanisms of alcohol-induced defects in cognition and therapeutic strategies to mitigate these defects. Lastly, we will highlight the importance of studying brain regions as part of a brain network in delineating the mechanisms of alcohol-induced cognitive impairment which can aid in the development of therapeutics for such indication.

## Pet Imaging and Cognitive Performance in Alcohol Intoxication

Positron emission tomography imaging has been widely used as a minimally invasive method to assess neuronal synaptic activity and function. Relative hypometabolism of associative brain regions has been used to predict the rate of cognitive decline in Alzheimer’s disease distinct from that in normal aging (Silverman et al., 2008). Moreover, the magnitude of decline over a 2-year period for standard measures of memory correlated with hypometabolism in the parietal and temporal lobes, and in the cingulate cortical regions (Small et al., 2000; Silverman et al., 2008). An early autoradiographic study in rodents showed that while 1 g/kg body weight (bw) alcohol decreased brain metabolic activity, 0.25 g/kg increased the activity and 0.5 g/kg had very minimal effect (Williams-Hemby and Porrino, 1994). However, very few studies have been reported in alcohol and brain glucose metabolism using PET imaging. In one study, local cerebral metabolic rate was studied using PET and ^18^FDG in fourteen chronically alcohol-dependent patients and eight normal healthy subjects (Gilman et al., 1990). The patients were studied after a 45 day duration of abstinence and at least 27 days after the initiation of detoxification. The study showed hypometabolism in the frontal cerebral cortex in alcoholic dependent patients regardless of showing clinical signs of alcoholic cerebellar degeneration (ACD). Those subjects showing cerebellar degeneration exhibited hypometabolism in the superior cerebellar vermis in addition to the frontal cerebral cortex. The severity of the clinical neurological impairment significantly correlated with the degree of hypometabolism in the superior cerebellar vermis and the medial frontal cerebral cortex. The correlation between clinical symptoms and hypoactivity in the cerebellar vermis was restricted to ACD patients while the hypometabolism in the medial frontal cortex correlated with alcoholic patients with and without ACD.

In another study using PET and ^11^C-glucose, 12 healthy male subjects and nine male alcoholic inpatients who had been kept from alcohol and drugs for 4 weeks were studied (Wik et al., 1988). The alcoholics exhibited a 20–30% reduction in glucose metabolism in the cortical and subcortical regions. The distribution of the relative regional metabolic rates showed significant decreases in the parietal cortices suggesting that the parietal lobe was affected in alcoholics. However, there was no obvious relationship observed between the clinical characteristics and the regional metabolic rates. In another study, the effect of alcohol on the human brain was tested in non-alcoholic healthy subjects and in alcoholic subjects with acute oral administration of 1 g/kg alcohol 20–40 min before ^18^FDG administration. Alcoholic subjects were kept sober for 10–15 days prior to study. PET scans were obtained 1 h post alcohol intake at time intervals of 1, 2, and 8 min for a total of 14 scans. Regional metabolic rates were then calculated from the 8 min scan taken 35 min post ^18^FDG injection. The study showed that acute alcohol intoxication inhibited both cortical and cerebellar glucose metabolism with inhibition being more pronounced in the alcoholic subjects (Volkow et al., 1990). Subjective sense of intoxication and motor impairment were, however, comparable between groups. However, from this study it is not clear how much acute intoxication contributed to these effects. A subsequent study with low dose alcohol in healthy control subjects showed that acute oral administration of both 0.25 g/kg and 0.5 g/kg significantly reduced brain glucose metabolism 40–50 min after alcohol intake, but the responses differed between the two doses (Volkow et al., 2006). While 0.25 g/kg reduced metabolism in the cortical regions, 0.5 g/kg dose showed reduction in the cortical regions as well as the subcortical regions, i.e., cerebellum, mesencephalon, basal ganglia and the thalamus. Alcoholic effects on cognitive performance were only minimal from placebo vs. ethanol as well as between the two doses. In a rat model, brain glucose metabolism was measured using ^18^FDG-PET after 45 min of radiotracer uptake at basal and after acute administration by intraperitoneal injection of 1.5 g/kg alcohol (Gispert et al., 2017). Alcohol significantly reduced global brain glucose metabolism with the reduction being more pronounced in the parietal cortices and cerebellum. From these earlier studies, it was clear that acute alcohol intoxication causes reduction in brain activity either globally or regionally, but whether or not such reduction correlated with cognitive impairment was not conclusive.

Recently, we conducted a study using a mouse model of binge alcohol exposure and measured brain glucose metabolism and cognitive performance in the same cohort of mice (Jacob et al., 2019). Our model consisted mice, each receiving a continuous intravenous administration of a bolus dose of alcohol (1.5 g/kg) referred to a bolus infusion for over a 20 min period. After recovery from anesthesia and bolus infusion for 30 min, alcohol was delivered continuously at 300 mg/kg/h for 15 h in awake mice using a mouse harness that allowed free movement of mice in cages. The BAC in this model reached to 37–38 mM (0.17%) 1 h after the bolus infusion. Using this mouse model of alcohol, we examined the effect of alcohol in brain glucose metabolism and cognitive performance using ^18^FDG-PET and behavioral tasks, respectively (Jacob et al., 2019). First, mice were subjected to behavior tasks and a microPET scan to assess basal behavior and brain glucose metabolism, respectively. After a recovery period, mice were subjected to alcohol exposure as described above. At 15 h later, the mice underwent the post-alcohol behavior tasks and microPET scans. The PET images were analyzed using a mouse version of statistical parametric mapping (SPM5; Wellcome Department of Imaging Neuroscience, Institute of Neurology, London, United Kingdom) using a paired *t*-test. When globally normalized standard uptake values (SUV) were compared between pre- and post-alcohol, several distinct brain regions with altered glucose metabolism were identified. The regions that showed decreases were agranular insular cortex, dorsal part; secondary visual cortex, lateral area; olfactory bulb, glomerular layer; entorhinal/perirhinal cortex; spinal trigeminal tract and spinocerebellar tract. Unlike prior human subject studies, there was no significant change in the global glucose metabolism between pre- and post-alcohol. It has been suggested that the global decrease observed in alcoholic patients in the prior human studies could be attributed to the medication they receive as a treatment during detoxification. Disulfiram, a widely used medication, has been shown to inhibit the metabolism of acetaldehyde and slows down glucose metabolism globally (Gilman et al., 1996). Nevertheless, our findings in the mouse model of acute alcohol are consistent with previous human studies showing decreased regional brain glucose metabolism after acute alcohol intoxication.

We and others have shown that acute alcohol caused reduction in brain activities in the cortical regions and subcortical regions including the temporal lobe (Volkow et al., 1990, 2008; Jacob et al., 2019). The medial temporal lobe consists of the hippocampus and the adjacent entorhinal and perirhinal cortex which are prominent regions required for cognition, including learning and memory (Squire et al., 2004). In animal models, the hippocampus has been shown to be involved in spatial learning and memory. Animals often use spatial information to organize and guide behavior in cognitive tasks (Matthews and Best, 1997). Moderate ethanol exposure (1.25 g/kg) has shown to impair spatial working memory when animals were tested on a radial arm maze (Gibson, 1985). This effect was extended to slightly lower doses of 0.5–1.0 g/kg (Givens, 1995). In another study with a more challenging spatial working memory task, information learned in a single working memory session when the animal was sober could be disrupted following an acute alcohol challenge and the observed memory impairment was dose dependent (Hoffmann and Matthews, 2001). Behavior tasks in our study included object place memory (OPM) and open field (OF) tasks. In rodents, OPM has been investigated in open field format where an animal explores two objects spontaneously (sample trial) and after a variable delay, is re-exposed to the same objects with the exception that one object has been moved to a different location (choice trial). A positive discrimination ratio indicates preferential exploration of a moved object which represents spatial memory (Faust et al., 2013). This task is sensitive to lesions in the hippocampus, anterior thalamus and cingulate cortex (Bussey et al., 2000; Warburton et al., 2000). In our study, alcohol exposure showed a significant decrease in OPM between pre- and post-alcohol exposure indicating impaired spatial memory (Jacob et al., 2019). Alcohol exposure also displayed lower exploration of the center of the field in OF task as compared to pre-alcohol (Jacob et al., 2019). In the OF task, the exploration of the center as opposed to periphery indicate increased anxiety and familiarization to the environment. These behavioral assessments indicate cognitive impairment after acute alcohol intoxication. Therefore, our study showed a strong relationship between hypometabolism in the temporal lobe and impairment in spatial cognition after acute alcohol intoxication. Additional cognitive responses in response to acute alcohol involving the hippocampus such as spatial reference memory, contextual learning memory, trace conditioning and spontaneous alteration have been elegantly reviewed previously and will not be addressed here (Van Skike et al., 2019).

## Mechanisms of Acute Alcohol Intoxication and Cognitive Impairment

### Mechanisms of Impaired Cognition

Several studies addressed the mechanism of acute alcohol and cognition in distinct brain regions, i.e., the hippocampus, amygdala and the cerebellum. Alteration of the hippocampal neurophysiology has been implicated as a mechanism in acute alcohol induced hippocampal–dependent learning and memory. One of the first and critical findings in the direct effect of alcohol in the hippocampal neurophysiology is that alcohol inhibits NMDA-activated ion currents in the hippocampus (Lovinger et al., 1989, 1990). Similar alcohol concentration also inhibits the hippocampal long term potentiation (LTP) (Blitzer et al., 1990; Zorumski et al., 2014). Hippocampal glutamate levels are also decreased with acute alcohol concentration that produces cognitive deficits (Shimizu et al., 1998). Acute alcohol significantly decreased hippocampal theta rhythm, an oscillatory hippocampal field potential that predicts learning in cognitive tasks (Zhang et al., 2016). The expression of immediate early genes such as *c-fos* is decreased in the hippocampus after acute alcohol intoxication (Ryabinin et al., 1995). Subsequent studies have indicated that alterations in acute alcohol-induced hippocampal neurophysiology have been due to a decrease in acetylcholine in the hippocampus and that treatment with cholinesterase inhibitors attenuate alcohol-induced spatial memory impairment (Gold, 2003; Gawel et al., 2016).

It is also known that alcohol potentiates GABA inhibition and inhibits glutamate excitation in the hippocampal brain regions indicating these molecular entities are potential mechanistic mediators for acute alcohol-induced cognitive impairment. Acute alcohol intoxication increases allopregnanolone, a potent GABAergic modulator, in a variety of brain regions including the hippocampus implicating the release of allopregnanolone as a potential mechanism of acute alcohol associated cognitive deficits (Harrison et al., 1987; Morrow et al., 1987; Tokunaga et al., 2003; Izumi et al., 2007; Ramachandran et al., 2015). Pretreatment with the 5α-reductase inhibitor, finasteride, reduces allopregnanolone levels and reduces impairment in hippocampal-dependent spatial memory (Morrow et al., 2004). However, finasteride impacts multiple neurosteroids rendering it unsuitable for pharmacological intervention (Van Skike et al., 2019). Genetic depletion of *Srd5*α*1*, the gene encoding the enzyme 5α-reductase-1, a necessary enzyme for the formation of allopreganolone, had reduced effects on components of the plus maze but the majority of alcohol’s effects had not been different from the wild type mice (Ford et al., 2015; Tanchuck-Nipper et al., 2015). Another genetic depletion model, GABA_A_ receptor δ knockout mice that reduces the sensitivity of neurosteroids in behavioral and hippocampal electrophysiological studies has been suggested as a candidate mechanism to reduce hippocampal dependent spatial memory impairment (Mihalek et al., 1999; Stell et al., 2003). Although various genetically modified mouse lines of GABA_A_ receptor isoforms were investigated, these studies indicate that GABA_A_ receptors do not mediate alcohol-induced cognitive effects in the hippocampus (Berry et al., 2009; Martin et al., 2011).

Targeting the NMDA receptor (NMDAR) has proven to be more successful in delineating the mechanism underlying alcohol-induced memory impairment. Alcohol conveys its effect by inhibiting NMDAR-mediated LTP (Morris et al., 1986; Lovinger et al., 1989). This inhibition is dependent on striatal-enriched protein tyrosine phosphatase (STEP) as alcohol does not inhibit NMDA receptor mediated excitatory postsynaptic currents (EPSCs) or block LTP in CA1 pyramidal neurons in STEP knockout mice (Hicklin et al., 2011). These studies suggest that STEP plays a prominent role in alcohol-induced fear conditioning impairment. Recently a mouse strain mutant GluN1 subunit which is less sensitive to the effects of alcohol has been generated (den Hartog et al., 2013; Zamudio-Bulcock et al., 2018) but the effect of alcohol on cognition has not yet been determined. Additionally, genetic differences have been observed, as in the case of aldehyde accumulation. Aldehyde dehydrogenase 2 knockout mice have shown increased sensitivity to alcohol-induced memory impairment in the Morris water maze and the radial arm maze compared to wild type mice (Quertemont et al., 2005; Jamal et al., 2012).

The amygdala is involved in emotional learning and memory in humans (LaBar and Cabeza, 2006). In animals, the amygdala and its circuits are important for fear conditioning, especially cued fear conditioning (Fanselow and Poulos, 2005; Kim and Jung, 2006; Maren, 2008). Although the evidence is somewhat controversial, acute ethanol exposure has shown to impact fear conditioned and emotional memories (Gould, 2003; Knowles and Duka, 2004; Gulick and Gould, 2008). Specifically, alcohol produces retrograde facilitation and anterograde impairment of emotional memories in both humans and in mice (Knowles and Duka, 2004; Gulick and Gould, 2008). Therefore, these studies caution against drinking to alleviate depression, anxiety or frustration because alcohol facilitates recall of emotional memories of events occurred prior to intoxication (Van Skike et al., 2019). The insular cortex, which takes up less than 2% of the cortical region and receives afferents from sensory thalamic nuclei, is connected to the amygdala and other limbic-associated cortical areas (Nieuwenhuys, 2012). The human insular cortex is composed of three concentrically arranged zones called the agranular, dysgranular and granular cortex. Altered function of the agranular insular cortex and its output to subcortical limbic regions have been shown to mediate alcohol intake in animal models of AUD (Jaramillo et al., 2016, 2018a,b). Studies on the effect of acute alcohol exposure on agranular insular cortex, however, has been limited. One study showed that alcohol inhibited electrically evoked NMDAR-mediated EPSCs in a concentration dependent fashion but alcohol had no effect on electrically evoked AMPAR-mediated EPSCs or spontaneous EPSCs in the agranular insular cortex pyramidal neurons (Shillinglaw et al., 2018). This study suggests that glutamate is uniquely sensitive to alcohol and that NMDAR-mediated processes in the agranular insular cortex may be disrupted by acute alcohol intoxication (Shillinglaw et al., 2018).

Although the primary function of the cerebellum is motor planning and execution, it has non-motor functions including cognition. The importance of cerebellar circuitry in classical eyeblink conditioning, a form of associative motor learning, has been well characterized in both humans and animals (Cheng et al., 2008; Sun, 2012). Alcohol’s effect on cerebellar learning appears to be dose-dependent in which the low doses facilitated on eyeblink-conditioned response while high doses impaired learning (Hernandez and Powell, 1986; Hernandez et al., 1986). Acute alcohol intoxication exerts a biphasic response where low doses increase and high doses inhibit the spontaneous activity of Purkinje cells (Chu, 1983). This effect mirrors the eyeblink-conditioned response observed in cerebellar learning. These studies collectively suggest that alcohol inhibits cerebellar learning by inhibiting various mechanisms of cerebellar synaptic plasticity (Van Skike et al., 2019).

### Mechanism of Hypometabolism and Impaired Cognition

The mechanism responsible for hypometabolism and subsequent impaired cognition has not been completely understood. As the brain is highly inter-connected with parallel processing systems, it is important to consider the brain as a network in studying the effect of acute alcohol intoxication on cognition. Brain imaging such as the PET allows the identification of different brain regions that functions together as a brain network which could be involved in acute alcohol-induced cognitive impairment. Conducting behavioral task assessments in conjunction with PET imaging will further allow us to directly correlate brain activity and cognition assessment. Only a few exclusive human studies have addressed the effect of acute alcohol intoxication in the whole brain and have attempted to correlate those with cognitive performance (Volkow et al., 1990, 2006). Due to the limited studies conducted with PET imaging in conjunction with cognition assessment, the human studies were not able to confirm a correlation between cognitive performance and brain metabolism in acute alcohol intoxication. Furthermore, the molecular targets in the brain of hypometabolism and acute alcohol intoxication have not been elucidated.

Based on this knowledge, we have investigated the role of a novel inflammatory mediator, extracellular cold inducible RNA binding protein (eCIRP) in acute alcohol-induced brain hypometabolism and its correlation with cognitive impairment. CIRP belongs to a family of cold shock proteins (Nishiyama et al., 1997) and recent work identified a novel function of CIRP, in that, upon cellular stress, it is released into the circulation and acts as a damage associated molecular pattern (DAMP) which promotes inflammation (Qiang et al., 2013). Thus, eCIRP functions as a stress protein and we reason that it could alter brain activity and cognition during excessive acute alcohol exposure. To test this notion, we exposed CIRP knockout mice and wild type mice to acute alcohol intoxication and assessed relative brain glucose metabolism with ^18^FDG-PET and behavioral assessment, i.e., OPM and OF tasks as described in the previous section (Jacob et al., 2019). In the SPM analysis of ^18^FDG-PET uptake, while relative brain metabolism decreased in the wild type mice in the limbic (entorrhinal/perirhinal cortices and the hippocampus) region, relative brain metabolism was less suppressed in the same region in CIRP knockout mice. Behaviorally, acute alcohol- exposed wild type mice were impaired in exploring a repositioned object in the OPM task and were more anxious in the OF task, whereas CIRP knockout mice were not impaired in either of these tasks. This study also showed a strong correlation between the changes in the relative metabolism and the changes in OPM from pre- and post-alcohol exposure in the fimbria of the hippocampus indicating a direct correlation between brain metabolism and cognition. We also observed a direct correlation between regions identified by the ^18^FDG-PET imaging; i.e., the fimbria of the hippocampus correlated with the cortical amygdala suggesting the involvement of a brain network in acute alcohol intoxication. This study suggests that eCIRP could be a critical mediator in hippocampal-dependent memory impairment. While the relative metabolic activity in the cerebellar vermis was decreased in the wild type mice, such activity was less suppressed in the CIRP knockout mice suggesting that CIRP could be involved in the impairment of cerebellar learning (Jacob et al., 2019). It was also striking to observe that the relative metabolic activity was decreased in the wild type mice after alcohol in the agranular insular cortex while the activity was less suppressed in the CIRP knockout mice. However, the mechanism and the significance of such findings in acute alcohol intoxication have not been determined. In alcoholics, studies using neuroimaging, physiological, neuropathological and neuropsychological approaches collectively indicate that the frontal lobes, limbic system and cerebellum are particularly prone to damage and dysfunction (Oscar-Berman and Marinkovic, 2007). It was interesting to observe that at least with neuroimaging, similar regions have shown hypometabolism in acute alcohol intoxication and that eCIRP could play a role in such defects.

## Therapeutic Interventions in Cognitive Impairment After Acute Alcohol Intoxication

Binge drinking or excessive acute alcohol drinking is a risk factor for developing AUD and most AUD patients binge drink. To date, only three drugs have been approved by the Food and Drug Administration (FDA) as treatments for AUD; Disulfiram, naltrexone and acamprosate. Disulfiram inhibits aldehyde dehydrogenase causing accumulation of acetaldehyde, a toxic intermediary metabolite of alcohol (Suh et al., 2006). Acetaldehyde build-up leads to symptoms such as nausea and vomiting which deter the user from further alcohol intake. Naltrexone is an opioid receptor antagonist which attenuates the rewarding effect of alcohol (Swift et al., 1994; Jarjour et al., 2009). Acamprosate prevents the craving for alcohol by modulating the imbalance between glutamatergic and GABAergic systems (Kiefer and Mann, 2010; Spanagel et al., 2014). The clinical uses of these drugs are primarily designated for alcoholic patients who have completed alcohol withdrawal and are committed to abstinence (Kahan et al., 1995; Krishnan-Sarin et al., 2008; Garbutt, 2009). However, contraindications exist for all three of these drugs and all have limitations for use. Although there are over 30 compounds in Phase I and Phase II clinical trials in the United States for AUD, none have yet emerged as an effective therapy for cognitive impairment in AUD. A few compounds have shown potentials in preclinical studies and one case report. For instance in a case report, Donepezil, a cholinesterase inhibitor used clinically to ameliorate memory-related cognitive deficits in Alzheimer’s disease, given to one alcoholic patient who presented with cognitive impairment without any typical findings of Alzheimer’s disease showed improvement in cognitive functions (Kim et al., 2004). A great body of literature exists on preclinical data on investigational drugs that may have therapeutic potential in AUD. Several compounds targeting the neuropeptide systems, epigenetic modifications and neuroinflammatory/neuroimmune modulators have been implicated as therapeutics in preclinical studies of acute alcohol (Ch’Ng and Lawrence, 2018). In the case of neuropeptide systems, ghrelin and neurokininin 1 receptor antagonists, oxytocin and glucagon-like-peptide 1 receptor agonists have been reported (Jerlhag et al., 2009; Peters et al., 2013; Schank et al., 2013; Vallof et al., 2016). Histone deacetylase inhibitors and DNA methyltransferases that modulate the dysregulated epigenetic mechanism have been implicated (Sakharkar et al., 2014; Jeanblanc et al., 2015; Simon-O’Brien et al., 2015; Qiao et al., 2017). Phosphodiesterase inhibitors and P2X4 receptor positive allosteric modulators have been considered in the context of neuroinflammatory/neuroimmune modulators (Hu et al., 2011; Yardley et al., 2012, 2014; Franklin et al., 2015). In terms of neuroinflammatory modulators, it has been demonstrated that chronic alcohol intake results in neuroinflammation and that pharmacotherapies targeting neuroinflammatory mediators could be neuroprotective and mitigate alcohol induced cognitive dysfunction. Peroxisome proliferator activated receptors (PPARs) and Toll like receptors (TLR) are implicated in alcohol related neuroinflammation (Ch’Ng and Lawrence, 2018). In this regard, previously we have shown that eCIRP mediates its effects via the TLR4/myeloid differentiation 2 (MD2) complex and that a small peptide inhibitor of eCIRP, C23, binds to this complex with high affinity (Qiang et al., 2013). The C23 peptide has shown to be protective in several murine models of stress conditions (McGinn et al., 2018; Denning et al., 2019; Zhang et al., 2017, 2018). It can be speculated that blocking eCIRP’s effect with C23 could prevent cognitive impairment in AUD. The effect of C23 in our rodent model of acute alcohol has not been determined.

## Future Research and Perspectives

**FIGURE 1:**
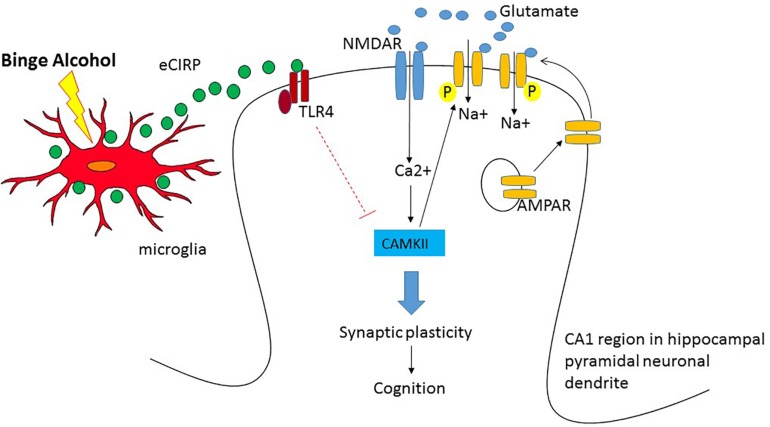
Potential mechanism of hypometabolism and impaired cognition after acute alcohol intoxication in the pyramidal neurons of the CA1 region in hippocampus. When glutamate is released from the pre-synaptic terminal, NMDA receptor (NMDAR), the inotropic channel, opens and allows the entry of Ca^2+^ into the post-synaptic terminal and depolarizes the cell. The entry of Ca^2+^ activates Ca/Calmodulin Kinase II (CAMKII) and facilitates phosphorylation and trafficking of AMPAR into the cell membrane causing sustained depolarization and long term synaptic potential leading to cognition. Upon binge alcohol exposure, cold inducible RNA binding protein (CIRP) released from microglia binds to Toll like receptor 4 (TLR4) and the activation of the TLR4 pathway presumably decreases the expression of CAMKII and attenuates synaptic plasticity leading to cognitive impairment.

Early human studies using ^18^FDG-PET suggest that alcohol intoxication causes brain hypometabolism in the cortical and subcortical regions (Volkow et al., 1990, 2008). In a subsequent rat model with intraperitoneal injection of acute alcohol, the parietal region and the cerebellum showed hypometabolism (Gispert et al., 2017). In our study using a mouse model of acute alcohol intoxication using intravenous infusion of alcohol, we observed significant reduction in the brain glucose metabolism in the temporal lobe and the secondary visual cortex as well as cerebellum (Jacob et al., 2019). Taken together these studies strongly demonstrated that high doses of acute alcohol caused reduction in brain metabolic activity. However, the data were inconclusive from the earlier human studies whether the reduced brain glucose metabolism led to cognitive impairment in high acute alcohol. Using behavior analysis in conjunction with ^18^FDG-PET, our study is the first to show correlation between brain glucose metabolism in the hippocampus and a hippocampal-dependent behavioral task, i.e., OPM task demonstrating reduction in brain glucose metabolism leading to cognitive impairment in acute alcohol intoxication. We observed similar reduction in neuronal activity in the cortical amygdala, cerebellum and the agranular insular cortex. Since brain is highly inter-connected and functions as a network, it is possible that the lack of cross talk among these regions are responsible for cognitive impairment in acute alcohol intoxication. Furthermore, our study using ^18^FDG-PET in conjunction with OPM task suggest eCIRP as a potential mechanistic mediator in cognitive impairment during acute alcohol intoxication (Jacob et al., 2019). How eCIRP causes brain hypometabolism and impaired cognition has not been completely elucidated. A potential mechanism of eCIRP’s effect on the brain leading to impaired cognition is shown (Figure 1). First, since eCIRP has shown to be a stress protein (Qiang et al., 2013), we reasoned that binge alcohol drinking could release eCIRP from the immune cells in the brain such as the microglia and act on the neurons to mediate its effect. In this regard, we have shown that in BV2 cells, a mouse microglia cell line, acute alcohol exposure at high doses released eCIRP into the culture medium suggesting microglia could be responsible for eCIRP release in the brain (Rajayer et al., 2013). Secondly, how eCIRP mediates hippocampal-dependent memory impairment as observed in the OPM task has also not yet determined. The best understood mechanism of how memories are formed is the glutamate mediated neurotransmission or synaptic plasticity (Malenka, 1991; Citri and Malenka, 2008; Martin et al., 2000). Synaptic plasticity consists of the activity-dependent modification of the strength of synaptic transmission at the pre-existing synapses which plays central role in the brain’s capacity to incorporate transient experiences to persistent memory traces. Repetitive activation of excitatory synapses in the hippocampus causes the potentiation of synaptic strength which could last for hours to even days. Alcohol’s effect on both acute and long term memory are mediated by glutamatergic neurotransmission (Chandler et al., 1998; Lovinger and Roberto, 2013). Acute alcohol exposure has shown to induce aberrant synaptic plasticity and impaired behavior (Ma et al., 2017). The most extensively studied form of synaptic plasticity is LTP observed in the pyramidal neurons of the CA1 region of the hippocampus (Citri and Malenka, 2008). Therefore it is plausible that eCIRP released from microglia present in the hippocampus acts directly on the pyramidal neurons and decreases LTP of the CA1 hippocampal region. Alternatively, alcohol suppression of LTP in the hippocampus could indeed be a consequence of alteration in the connected brain regions as part of an extended neural network ultimately resulting in impaired cognition. Future studies are needed for dissecting the actual mechanism by which eCIRP causes hypometabolism and impaired cognition. We strongly recommend that future mechanistic studies should be performed bearing in mind that the brain functions as a network with parallel processing systems. Thus, conducting mechanistic studies with PET imaging in conjunction with behavioral tasks, could aid in the development of effective therapeutics for AUD in the future.

## Author Contributions

AJ gathered the literature, drafted and revised the manuscript. PW provided the resources and critically reviewed the manuscript.

## Conflict of Interest

The authors declare that the research was conducted in the absence of any commercial or financial relationships that could be construed as a potential conflict of interest.
